# Community-based maternal and perinatal death surveillance and response: a comparative case study of implementation realities from humanitarian contexts

**DOI:** 10.1186/s12889-025-24440-2

**Published:** 2025-10-01

**Authors:** Meighan Mary, Hannah Tappis, Elaine Scudder, Andreea A. Creanga

**Affiliations:** 1 International Health Department, Johns Hopkins Bloomberg School of Public Health, 615 N Wolfe St, Baltimore, MD 21205 USA; 2 International Center for Maternal and Newborn Health, Johns Hopkins Bloomberg School of Public Health, Baltimore, MD USA; 3 Center for Humanitarian Health, Johns Hopkins University, Baltimore Maryland, USA; 4 Jhpiego, Baltimore, MD USA; 5 International Rescue Committee, Washington, DC USA; 6 Department of Gynecology and Obstetrics, Johns Hopkins School of Medicine, Baltimore, MD USA

**Keywords:** Maternal mortality, Neonatal mortality, Surveillance, Death review, Maternal and perinatal death surveillance and response (MPDSR), Humanitarian

## Abstract

**Background:**

Implementation of community-based Maternal and Perinatal Death Surveillance and Response (CB-MPDSR) in crisis-affected settings offers an opportunity to adapt humanitarian programming and mount solutions to directly improve maternal and neonatal health among those most in need. This study aimed to understand factors that influence implementation of CB-MPDSR approaches across diverse humanitarian contexts.

**Methods:**

A comparative qualitative case study was conducted in December 2021-July 2022 to assess CB-MPDSR implementation in 4 diverse humanitarian settings: Cox’s Bazar (CXB) refugee camps, Ugandan refugee settlements, South Sudan, and Yemen. A scoping review and 39 individual or group semi-structured key informant interviews were conducted. Thematic content analysis was employed to understand the adoption, penetration, and fidelity of CB-MPDSR approaches and elucidate cross-setting learning.

**Findings:**

Adoption of CB-MPDSR varied: refugee contexts in CXB and Uganda had well-established systems involving active pregnancy and mortality surveillance and verbal autopsy. In Yemen, implementation was reliant upon passive reporting mechanisms, while implementing partners in South Sudan employed a mix of strategies. Financial, human resources, and socio-cultural dynamics significantly limited implementation, especially the notification and review of perinatal deaths. Strategic engagement of community stakeholders was employed to improve participation and transparency between communities and health systems; however, community trust in the humanitarian health system remains an unresolved issue.

**Conclusions:**

CB-MPDSR offers insights into important systemic and cultural factors contributing to mortality within crisis-affected settings. Our results call for more research investment in understanding how to effectively adapt CB-MPDSR and development of operational guidance to assist humanitarian actors in introducing or bolstering CB-MPDSR approaches, so as to support a system reflective of complex realities faced by these diverse and mobile communities.

**Supplementary Information:**

The online version contains supplementary material available at 10.1186/s12889-025-24440-2.

## Introduction

Since 2004, global stakeholders have recommended the implementation of mortality review interventions to improve health service delivery and reduce maternal and perinatal mortality in low- and middle-income countries (LMICs) [1–3]. In addition, guidelines have been developed for building comprehensive maternal and perinatal death review and response (MPDSR) systems focused on death notification, report, review, and response at facility and community levels [4–6]. Evidence about implementation of MPDSR and related death review interventions presents a plethora of implementation challenges within health facilities [7–11], and reinforces recommendations for a phased introduction of MPDSR ensuring effective implementation in secondary and tertiary-level health facilities before scaling to lower levels of the health system and within communities where implementation may involve a gamut of actors, structures, and systems with and without linkages to the health sector [4, 5]. As such, learnings from implementation of community-based MPDSR (CB-MPDSR) approaches are limited, with most literature concentrated on descriptions of community involvement and engagement in MPDSR and mortality review interventions in relation to facility-based death reporting and review [7].

Nonetheless, as global trends of forced displacement, conflict, and complex emergencies continue to break records, the magnitude of populations without access to health services and who are only served by weakened or fractured health systems is rising [12]. In these crisis-affected contexts where a vast number of births and maternal and perinatal deaths often are undocumented within communities, CB-MPDSR approaches can provide action-oriented information on key drivers of maternal and perinatal mortality to improve humanitarian response. Yet, despite increasing acknowledgment of the importance and value of CB-MPDSR approaches in settings affected by crises [13–15], evidence on key implementation outcomes[16] from these contexts remains scant [17].

A scoping review of evidence on the implementation of MPDSR and related death review interventions in humanitarian contexts from 2016 to 2023[17], only identified 12 programs implementing CB-MPDSR approaches [15, 18–29] in 8 of 29 countries with a humanitarian funding appeal issued by the UN Office for Coordination of Humanitarian Affairs (UN OCHA) in 2023 [30]. Challenges in implementing CB-MPDSR approaches related to limited financial and human resources and strained dynamics with communities were reported across contexts [17]. Nonetheless, gaps in understanding how to effectively implement CB-MPDSR are still considerable and call for further investigation that employs an implementation science lens to assess how best to adapt and strengthen CB-MPDSR approaches in crisis-affected contexts. In efforts to bolster the current evidence base, this study aimed to synthesize key implementation outcomes and understand factors that influence implementation of CB-MPDSR approaches in diverse humanitarian crises.

## Methods

### Study design

A qualitative comparative case study was conducted to examine implementation of CB-MPDSR in four contexts: Rohingya refugee camps in Cox’s Bazar (CXB) Bangladesh, Ugandan refugee settlements, and humanitarian crises in South Sudan and Yemen. Semi-structured individual or group key informant interviews were conducted from December 2021-July 2022 and triangulated with information collected on case-specific CB-MPDSR approaches via scoping review (results published elsewhere) [11]. The research protocol was reviewed by the Johns Hopkins Bloomberg School of Public Health Institutional Review Board (IRB18926) and classified as not human subjects research.

Cases were defined using two criteria: (1) humanitarian contexts with a 2021 United Nations humanitarian or refugee response plan and (2) one or more CB-MPDSR approaches reported in a programmatic landscape analysis of MPDSR in humanitarian contexts [31]. Expanding upon the WHO definition of community-based surveillance (CBS) [13], CB-MPDSR approaches were defined as the notification, reporting, and/or review of maternal and perinatal deaths by community actors, including but not limited to community leaders, community members, and skilled and unskilled community health worker cadres responsible for providing health services or education at the community level. Diversity in geographical region and humanitarian setting (e.g., refugee, internally displaced, or mixed) were prioritized for case selection. Key characteristics of each selected case are outlined in Table 1.

**Table 1:** Case characteristics

Characteristics	Bangladesh	Uganda	South Sudan	Yemen
*Case context*				
Case focus	Cox’s Bazar Rohingya refugee camps ^a^	Uganda refugee settlements ^a^	National	National
WHO Region	Southeast Asia	Africa	Africa	Eastern Mediterranean
World Bank Income Classification FY2023 [32]	Lower middle	Low	Low	Low
World Bank Fragility Classification FY2023 [33]	–	–	Conflict	Conflict
Consecutive years of humanitarian and/or refugee response plans[34]	7	6	13	14
People in need of humanitarian assistance 2023[12]^b^	1.5 M (1%)	1.5 M (3%)	7.8 M (84%)	21.6 M (73%)
*National maternal and perinatal health outcomes*				
Maternal mortality ratio[35]^c^	123 (89–174)	284 (191–471)	1223 (746–2009)	183 (120–271)
Neonatal mortality rate[36]^d^	16 (14–18)	19 (13–28)	40 (12–105)	28 (13–61)
Stillbirth rate[37]^e^	21 (16–26)	15 (14–16)	26 (16–42)	23 (16–34)

Abbreviations: FY, Fiscal Year; WHO, World Health Organization;

^a^Refugee camps are plots of land with temporary homes made available to host refugees. Refugee settlements are also made available to host refugees but resemble “open” villages rather than restricted camps

^b^M: million; Reported as n (% of total population)

^c^2020 United Nations Maternal Mortality Estimation Inter-Agency Group Maternal Mortality estimates reported as number of maternal deaths per 100,000 live births; Values in parentheses represent 80% uncertainty intervals

^d^2021 United Nations Inter-Agency Group for Child Mortality Estimation Neonatal Mortality estimates reported as number of neonatal deaths per 1000 live births; Values in parentheses represent 95% uncertainty intervals

^e^2021 United Nations Inter-Agency Group for Child Mortality Estimation Stillbirth Rate estimates reported as number of stillbirths per 1,000 births; Values in parentheses represent 90% uncertainty intervals

### Study setting

The study was undertaken in four diverse humanitarian contexts, with varying health and political dynamics.

#### Refugee settlements in Uganda

Considered to have some of the most progressive refugee policies globally [38, 39], Uganda hosts over 1.5 million refugees and asylum seekers—and notably, the most refugees in Africa [40]. The forced migration of Sudanese to Uganda began in the mid-1980s was followed by subsequent waves of asylum seekers and refugees from Somalia, Rwanda, Burundi, Eritrea, Sudan, and Ethiopia over the past three decades. Most recently, Uganda has experienced large influxes of refugees from South Sudan and the Democratic Republic of Congo (DRC). The vast majority of refugees (94%) reside in thirteen settlements alongside Ugandan communities across thirteen districts in North and South-west regions of Uganda [40, 41]. Refugee settlements are largely supported by UNHCR and resemble “open” villages (rather than restricted camps), given that refugees have the right to migrate freely, work, establish businesses throughout the country [38, 39]. Refugees can also access health and education services at the same establishments as Ugandan nationals. With an integrated national public health and refugee health system since the early 2000’s, over 76% of health facilities in refugee hosting districts are accredited by the Ministry of Health [40]. Historically, refugees in Uganda have had better access to higher quality, well-equipped and staffed health facilities compared to national populations due to the humanitarian response, and as such, integration with the national system was encouraged as an equity measure to improve health care for Ugandans [42]. Nevertheless, even with integration efforts, health services are still largely supported and managed by international or local non- governmental organizations (NGOs) serving as implementing partners for UNHCR and other bilateral agencies in collaboration with the Ugandan Ministry of Health.

#### Rohingya refugee camps in Cox’s Bazar, Bangladesh

For almost a half of a century, targeted persecution, and violence against the Rohingya people in the Rakhine State of Myanmar has led to repeated large-scale displacement across the border into Bangladesh, the largest and most recent displacement following violence in August 2017. Over 950,000 Rohingya refugees are registered in a vast network of 33 camps in the Ukhiya and Teknaf sub-districts of CXB, Bangladesh [43]–the world’s largest refugee settlement. Humanitarian response is coordinated by the Government of Bangladesh, with the Refugee Relief and Repatriation Commission responsible for direct management and oversight, with guidance from the Strategic Executive Group, co-chaired by United Nations High Commissioner for Refugees (UNHCR) and the International Organization for Migration (IOM). At the field level, the Inter-Sector Coordination Group (ISCG) with membership from UN agencies, international and Bangladeshi non-governmental organizations, and donors, leads response in Cox’s Bazar. The vast majority of Rohingya households are moderately to highly vulnerable and significantly dependent upon humanitarian aid [44], with little access to livelihood opportunities. Health services are supported and/or delivered by over 70 health sector partners through an extensive network of registered health facilities [45]. Weak shelter infrastructure, limited access to water, sanitation, and hygiene, and geographic constraints of the crowded camps exacerbate challenges in the humanitarian response [44].

#### South Sudan

The complex humanitarian crisis in South Sudan is rooted in protracted insecurity, ignited by decades of civil war and ethnic violence, and ravaged by climate change, food insecurity, and public health crises. In 2023, approximately 76% of the population, including 2.2 million women and 4.9 million children, were estimated to need humanitarian assistance along with over 200,000 people in Abyei Administrative Area (the disputed territory between Sudan and South Sudan)[46]. Approximately 2.2 million people are displaced within the country and another 2.3 refugees are hosted in neighboring countries. Recently, intercommunal violence ensued in the Abyei Administrative Area and 7 out of 10 states, including attacks by armed cattle keeps, revenge attacks, abductions, and looting [46]. South Sudan is also the most dangerous crisis for humanitarian workers—in 2022, nine aid workers were killed in duty [47]. Sustained violent conflict has crippled the health system, rendering the health sector significantly dependent upon external support. Destroyed and looted health facilities, health worker shortages, and severe underfunding directly impede health service delivery further deteriorating public health [48–52]. In 2018, only 44% of the population was estimated to reside within 5 km of a health facility [53].

#### Yemen

After more than eight years of conflict combined with economic collapse and environmental disasters, Yemen is one of the world’s most complex humanitarian crises with the fifth largest internal displacement crisis globally [54]. During recent decades, political tensions have erupted into escalations in conflict. In 2022, the UN brokered a six-month truce resulting in no airstrikes, military operations, and significant reductions in displacement and civilian casualties. However, since its expiry, no agreement has been reached and fears of returned violence are ubiquitous [55]. Despite intermittent reduction of violence in 2022, the conflict is one of the deadliest for civilians, earning Yemen the ranking of second least peaceful country in the world according to the Global Peace Index [56]. In 2023, 21.6 million people, an estimated 66% of the population, were in need of humanitarian aid [55]. Prolonged armed violence along with ongoing climate shocks and disease outbreaks have had a devastating impact on civilians—over 80% of the population struggles to access food, water, and health care [57]. The weakened health system is near collapse, with less than half of health facilities functioning and an estimated 42% of the population having to travel more than an hour to reach a functioning (or partially functioning) hospital. Health service delivery is significantly reliant upon humanitarian support, with fluctuating levels of humanitarian funding an ominous threat to the closure of health facilities and/or disruption of essential health services.

### Sample

Key informants in each case were identified and recruited using purposive and snowball sampling. The research team consulted recent MPDSR programmatic landscape analysis findings [31] and collaborated with SWG members and representatives from the Interagency Working Group for Reproductive Health in Crises (IAWG) to identify all agencies implementing or supporting CB-MPDSR approaches. At least one key informant from each identified agency was invited to participate. Key informants were eligible to participate if they were 18 + years of age, currently engaged in supporting the implementation of CB-MPDSR approaches, and willing to participate in an audio recorded interview.

To achieve saturation in key themes, a sample of 5–15 participants per case was estimated. Across the four cases, 39 interviews were conducted with a final sample of 45 participants (Table 2). Participant representation varied by context, reflecting the diversity in humanitarian agencies engaged in supporting CB-MPDSR approaches. All identified agencies implementing or supporting CB-MPDSR approaches were represented in the final sample; none of the invited participants refused to participate.

**Table 2:** Sample size

	Cox’s Bazar Rohingya refugee camps	Uganda refugee settlements	South Sudan	Yemen	Total
Interviews	13	13	8	5	39
Participants^a^	13	15^b^	12^c^	5	45
*Participant sex:*					
Female	6	4	2	5	17
Male	7	11	10	0	28
*Participant representation:*					
UN Agency	10	4	2	2	18
Ministry of Health	0	1	1	1	3
INGO	2	7	5	0	14
Local NGO	0	0	4	0	4
Facility MPDSR focal point	0	2	0	2	4
Other	1	1	0	0	2
*Role:*					
Supported implementation	6	3	3	1	13
Directly involved in implemention	7	12	9	4	32

Abbreviations: INGO, International Non-Governmental Organization; MPDSR, Maternal and Perinatal Death Surveillance and Response; NGO, Non-Governmental Organization; UN, United Nations

^a^The differential between the number of interviews and number of participants in some cases reflects the option for group interviews

^b^One interview had 3 participants

^c^Three group interviews were conducted: one interview with 3 key informants and two interviews with two key informants

### Data collection

Identified key informants were invited via email to participate in semi-structured interviews; email invitations outlined the study aims, introduced the study interviewer (MM), and included a copy of the oral consent script and a study brief for reference. Key informants did not have any relationship established with the researchers prior to the study nor were they aware of the interviewer’s background. To accommodate schedules and the limited bandwidth of many humanitarians, individual or group interviews were offered. All interviews were conducted virtually by MM via Zoom in English, with the support of live Arabic interpretation for two interviews with key informants in Yemen. Interviews commenced with an informed consent script and lasted 45–90 min. All key informants orally consented to participation and recording of interviews. To maintain confidentiality, only participants were present during the interviews.

Interviews were facilitated using a piloted semi-structured interview guide (Supplementary Information: Annex 1) to understand key implementation outcomes of CB-MPDSR approaches (i.e., adoption, penetration, and implementation fidelity) along with factors that influence implementation. Implementation outcomes were selected a priori due to their saliency in the study contexts. Among Proctor et al.’s conceptual model of implementation research [16, 58], adoption, penetration, and fidelity outcomes are most salient for contexts in early to mid (1–5 years) implementation stages. Table 3 outlines the definitions of each outcome and associated thematic constructs. In addition, available documentation related to the reported CB-MPDSR approaches (i.e., policies, guidelines, reports, etc.) was requested from each key informant. Relevant information was also collected via a scoping review of peer-reviewed literature and grey literature electronically sourced from humanitarian web-portals (e.g., ReliefWeb and Humanitarianresponse.info) [11].

**Table 3:** Key Implementation Outcomes and Thematic Constructs

Implementation outcomes	Definitions^a^	Thematic constructs
Adoption	How CB-MPDSR approaches are intended to be implemented: The uptake of CB-MPDSR approaches from the organizational or implementer perspective	Governance structures Policy adoption Intended implementation processes: notification, report, verbal and/or social autopsy^a^, and response Implementation readiness: data systems and tools, training, and capacity building
Penetration	How CB-MPDSR approaches are scaled: The integration of CB-MPDSR approaches within communities and health systems in humanitarian settings	Scale of implementation Linkages with facility-based MPDSR interventions Integration with national MPDSR systems
Fidelity	How CB-MPDSR approaches are “actually” put into practice: The degree to which CB-MPDSR approaches were implemented as intended, according to local, national, or international guidelines or action plans	Adherence to CB-MPDSR cycle or protocol Quality of reporting and review Implementing actor responsiveness Community engagement

^a^Definitions of study implementation outcomes were adapted from Proctor et al.’s conceptual model for implementation research [16, 58]

^b^Verbal autopsy is defined as “an approach to determine levels and medical causes of death for people whose deaths are not registered.” Social autopsy is defined as a “method that focuses on social and health systems determinants of outcomes, providing supplementary information to that on medical cause of death.” Each method can be employed individually. However, when combined, they provide a comprehensive understanding of the contributing factors and causes of deaths [59]

### Analysis

Interview audio-recordings were transcribed in English by an external agency and verified by the research team. All transcripts and related documentation obtained via desk review were analyzed by MM using Dedoose qualitative software (version 8.3.45). A priori codes were derived from the thematic constructs related to each implementation outcome (Table 3) and analyzed using thematic content analysis to identify sub-codes and emergent themes within and across cases [16, 60].

Data were synthesized to generate case descriptions outlining key findings related to the adoption, penetration, and fidelity of CB-MPDSR implementation. Complementary data sources from the scoping review were triangulated with interview findings to understand the adoption of CB-MPDSR approaches in each case. Each in-depth case description was shared with participating key informants for validation [61]. To understand factors influencing implementation of CB-MPDSR approaches across humanitarian settings, cross-case synthesis examined commonalities and divergence in two emergent themes using both deductive and inductive analysis [61]: (1) implementation inputs (i.e., financial and human resources) and (2) socio-cultural dynamics (e.g., interplay between cultural, social, and contextual factors and implementation of CB-MPDSR approaches).

### Reflexivity statement

MM is a white female PhD researcher from the United States with over a decade of experience in international maternal health. MM recognized how her social position and experience may have shaped the interview dynamics and interpretation of information shared about the implementation CBMPDSR within humanitarian contexts and practiced ongoing reflexivity throughout the data collection and analysis processes. During interviews, she actively sought to minimize power dynamics by establishing rapport with participants and practicing memoing to document interpersonal reflexivity [62]. Team meetings were also held to discuss emerging findings along with the research team’s positionality, personal biases, and the contextual dynamics.

## Results

### CB-MPDSR landscape

Implementation of CB-MPDSR interventions varied by type of setting (i.e., refugee camp, rural and urban settings). Contexts with refugee camps or settlements had more complex or comprehensive CB-MPDSR interventions in place, often including active pregnancy and all cause death surveillance systems (CXB and Uganda). They also reported longer-term and larger-scale implementation experience compared to South Sudan and Yemen whose CB-MPDSR approaches were being piloted mostly outside of refugee contexts. In South Sudan, multiple CB-MPDSR approaches were reported and while all interventions included community-based reporting, the subsequent conduct of social or verbal autopsy was variable. Table 4 details the differences in the implementation landscape of CB-MPDSR approaches by case.

**Table 4:** Characteristics of CB-MPDSR approaches and adoption constructs

	Cox’s Bazar refugee camps	Uganda refugee settlements	South Sudan	Yemen
Reported intervention types using CB-MPDSR approaches	MDSR	MPDSR	Three intervention types reported: (1) MPDSR (2) MDSR (3) Maternal Death Surveillance	MDSR
Implementation phase^a^	Early-mid	Early-mid	Early-mid	Early-mid
Setting^b^	Refugee camps	Refugee settlements	Refugee camps, urban, and rural	Urban and rural
Population covered	Refugees	Refugees and host communities	Refugees, IDPs, and host communities	IDPs and host communities
Governance structures	• UN agencies: UNFPA, UNHCR, WHO, IOM • IPs: PHD • Led by CHW Working group with support from SRH and EPI Working groups	• MoH • UN agencies: UNHCR, UNFPA, UNICEF • IPs: IRC, CARE, AVSI, Medical Teams International, African Humanitarian Action	• UN agencies: UNHCR • INGOs and IPs: CARE, SAVE, CRADA, GOAL, TADO	• MoPHP in South Yemen, Governorate of Hadrhamaut • UN agencies: UNFPA
Policy adoption	• No policies or guidelines established • Guidance on CB-MPDSR components included in standard operating procedures for MPMSR (2019)	• Guidance on CB-MPDSR components included in national MPDSR guidelines (2017) • Guidance on CB-MPDSR components included in UNHCR guidelines and protocols used by many partners	• No current MoH MPDSR guidelines • Guidance on CB-MPDSR components included in UNHCR guidelines and protocols used by many partners • National policies require registration of births and deaths that occur at both facility and community level	• Guidance on CB-MPDSR components included in Maternal Mortality Audit National Guidelines (2013)
*Intended implementation processes*				
Identification	• Active pregnancy and all-cause death surveillance led by CHWs	• Active pregnancy and all-cause death surveillance led by village health teams (VHT)	• Varies by partner • Mix of passive surveillance relying on community actors to identify deaths and active surveillance led by CHWs	• Passive surveillance: Funeral personnel (including body/corpse washers) and community members identify deaths of women of reproductive age; burial permit reports also reviewed • Active maternal death surveillance led by midwives
Notification or Reporting	• CHW supervisor notified of all-cause deaths on weekly basis by CHW • All death reports uploaded to EWARS database • WRA deaths identified within EWARS database	• Health facilities notified of all-cause deaths by VHT within 24–48 h of identification	• Varies by partner: • Community actors report deaths to CHWs or health facilities • Health facilities notified of deaths by CHWs	• WRA deaths reported to district focal points, who then notify surveillance officer or district RH directors • Health facilities notified of maternal deaths attended by community midwives within 24 h
Verbal autopsy	• CHW introduces midwife verbal autopsy teams to families • Midwives conduct verbal autopsies • Midwives reclassify deaths as maternal and non-maternal deaths and provide information to EWARS database	• Verbal autopsy conducted after 1–2 weeks by teams • Team composition varied by partner (e.g., representatives from the Refugee Welfare Committee Members, INGOs, facility administration, health providers, community leaders, and VHTs)	• Varies by partner; some are not conducting verbal autopsies	• Surveillance officer conducts verbal autopsies with support of RH director within 3 weeks of death
Response	• CHW supervisor and other stakeholders meet with reproductive health awareness groups and community leaders in each camp to discuss learnings and implications from verbal autopsies • Response plans developed by the multi-partner MPMSR sub-committee	• Community dialogues conducted with the communities to share observations from the verbal autopsies (and death reviews) and receive their feedback on barriers affecting timely seeking health care • Response plan also developed at facility-level	• Varies by partner; some are not implementing response components • One partner established community health committees to hold the health system accountable	• Findings from the community analyzed along with facility-based death reviews to mount response at facility and community levels
*Implementation readiness*				
Data systems and tools	WHO’s Early Warning Alert and Response System (EWARS) Adapted UNHCR tools	Dual reporting systems: UNHCR and MoH DHIS2 UNHCR tools or adapted tools used by national MPDSR system	• Dual reporting systems: DHIS2, IDSR, partner-specific systems • Tools vary by partner	• Customized e-system used for 6 months – discontinued due to funding shortages • Forms adapted from national Yemen guidelines
Training and capacity building	• UNHCR trains all CHWs annually on pregnancy surveillance, notification, and report maternal deaths • UNFPA has dedicated workshops and training for midwifery team on verbal autopsy	• 2017–2019: Training provided at national, regional, and district levels • Magnitude of VHT/CHW training is partner dependent	• Magnitude of training is partner dependent	• Initial orientation and awareness workshops held with key community actors • District focal points trained on system and procedures for notifying surveillance officers • Limited training for CB surveillance officers, reliance on prior experience

Abbreviations: CDC, Centers for Disease Control and Prevention; CB, Community-based; CB-MDSR, Community-based Maternal Death Surveillance and Response CB-MPDSR, Community-based Maternal and Perinatal Death Surveillance and Response; CHW, Community Health Worker; CRADA, Christian Recovery and Development Agency; DHIS2, District Health Information System 2; EPI, Epidemiology; EWARS, Early Warning Alert and Response System; IDP, Internally Displaced Persons; IDSR, Integrated Disease Surveillance and Response; IMC, International Medical Corps; INGO, International Non-Governmental Organization; IOM, International Organization for Migration; IPs, Implementing Partners; IRC, International Rescue Committee; MDSR, Maternal Death Surveillance and Response; MIHR, Momentum Integrated Health Resilience; MoH, Ministry of Health; MoPHP, Ministry of Public Health and Population; MPDSR, Maternal and Perinatal Death Surveillance and Response; PHD, Partners in Health and Development; RH, Reproductive Health; SAVE, Save the Children; SRH, Sexual and Reproductive Health; TADO, Touch Africa Development Organization; TBA, Traditional Birth Attendant; UN, United Nations; UNFPA, United Nations Population Fund; UNHCR, United Nations High Commissioner for Refugees; UNICEF, United Nations Children’s Fund; VHT, Village Health Team; WHO, World Health Organization; WRA, Women of Reproductive Age

^a^Implementation phases are defined as (1) early to mid: 1–5 years of implementation; and (2) mid to late: 5 + years of implementation

^b^Refugee camps are plots of land with temporary homes made available to host refugees. Refugee settlements are also made available to host refugees but resemble “open” villages rather than restricted camps

### Implementation outcomes

#### Adoption

Adoption of CB-MPDSR approaches (i.e., how they were intended to be implemented) was assessed in terms of governance, established implementation processes, and implementation readiness (Table 4). In all cases, CB-MPDSR approaches were primarily led or supported by UN agencies (i.e., UNHCR and UNFPA) and implemented by international and local NGOs. However, the MPDSR policy landscape varied across the selected cases. Where in place (Uganda and Yemen), national guidelines included guidance on CB-MPDSR approaches [63, 64]. In addition, UNHCR, WHO, and/or UNFPA technical guidelines were used for partner-specific CB-MPDSR interventions across all cases. Significant overlap in terms of scope (content and implementation strategy) was found between national and partner-specific technical guidelines in Uganda and Yemen; however, in both contexts, partner-specific guidance was more comprehensive. For example, in Uganda, UNHCR guidelines were more detailed in terms of data collection and conduct of death reviews compared to Ugandan national guidelines. In Yemen, UNFPA guidance focused on implementation of MDSR systems, whereas national guidelines discussed only maternal death audit procedures.

Across all contexts, CB-MPDSR approaches followed WHO guidelines [6], including 4 key steps: (1) death identification, (2) death notification and/or reporting, (3) verbal autopsy, and (4) response. However, partners implementing community-based maternal death surveillance in South Sudan do not conduct steps 3 and 4. Active surveillance systems were primarily used to identify deaths that occurred in the community in refugee contexts (CXB and Uganda). In these contexts, community health workers (CHWs) and village health teams (VHTs) regularly visited all households in their catchment area and were responsible for monitoring pregnancies and identifying and reporting deaths of all causes. Some CB-MPDSR approaches in South Sudan and Yemen also had active surveillance components, albeit to a lesser extent. For the most part, CB-MPDSR interventions were implemented with passive surveillance systems relying on community actors to identify and report deaths to CHWs or health facilities. Only Yemen reported use of community actors beyond the various CHW cadres to identify maternal deaths: funeral personnel (e.g., morgue attendants or dieners) were trained to notify deaths of women of reproductive age to district focal points. In Yemen, local burial permit reports were also reviewed regularly.

Verbal autopsies were conducted in all cases; they were undertaken by trained midwives or surveillance officers, in CXB and Yemen respectively; via teams of varying composition (e.g., health providers, health administration, NGO representatives) in Uganda; and using a mix of these approaches depending on the implementing partner in South Sudan. Verbal autopsies typically occurred 1–3 weeks after the reported death to respect cultural bereavement periods. CB-MPDSR approaches in CXB, Uganda, and Yemen included response mechanisms at both the community and health system levels. Response strategies varied in South Sudan; several implementing partners did not conduct response as part of their CB-MPDSR approach while others within refugee contexts disseminated findings and implications from verbal autopsies via community dialogue sessions and/or health awareness groups to develop and mount response strategies with buy-in at the community level.

Data systems and tools for CB-MPDSR approaches differed by context. In CXB, maternal death reporting was integrated into the WHO Early Warning Alert and Response System (EWARS). Adapted UNHCR reporting and verbal autopsy tools facilitated data collection. In Uganda, both Ministry of Health and UNHCR tools and reporting systems were used in parallel creating dual reporting systems. In South Sudan, tools and reporting systems varied by implementing partner, with dual reporting to the MoH and other partner-specific systems common. Reporting and verbal autopsy forms were adapted from national maternal death audit guidelines in Yemen and in the absence of a national health information system, a custom-made e-system was used for the first six month until funding shortages could no longer sustain it.

Beyond initial training and orientation provided at the launch of MPDSR and related death interventions in each context, the magnitude of training and capacity building offered for CB-MPDSR approaches varied widely by context and supporting agency or implementing partner. In CXB, UNHCR trained all CHWs annually on pregnancy surveillance including maternal death notification and reporting, and UNFPA provided intermittent workshops and training for the midwifery team responsible for verbal autopsies. In Uganda and South Sudan, UNHCR also provided regular training on CB-MPDSR approaches to actors implementing in refugee camps or settlements; however, the provision of training of community health worker cadres implementing CB-MPDSR approaches supported by other agencies or implementing partners was limited. In Yemen, key community actors and district focal points to whom they notify community maternal deaths only received training at program launch, while surveillance officers did not receive any formal training on CB-MDSR since it was deemed unnecessary due to their experience with other community-based surveillance efforts.

#### Penetration

**Fig. 1:**
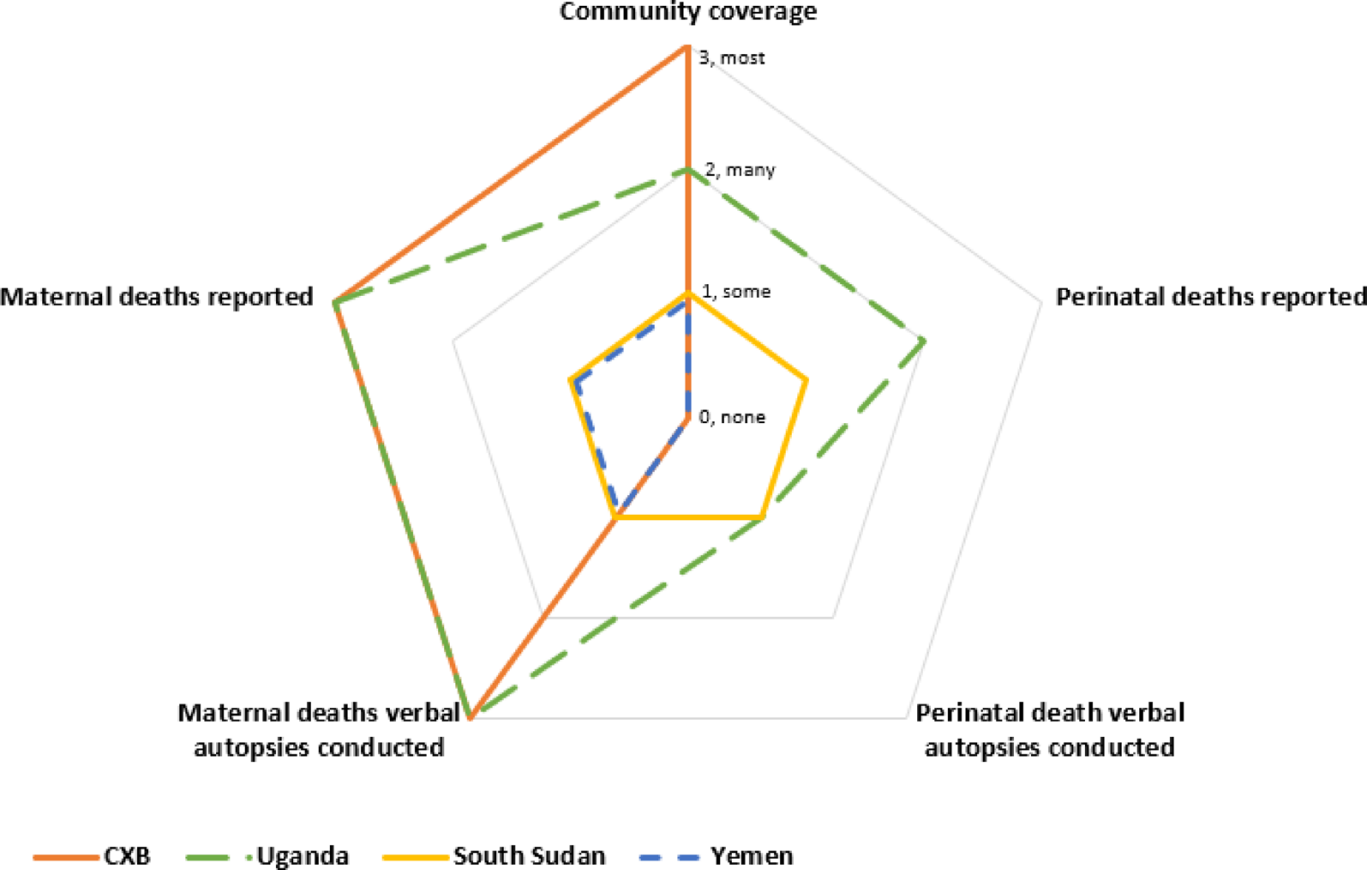
Scale of CB-MPDSR approaches by case Figure 1 represents a qualitative appraisal of the scale of community-based maternal and perinatal death reporting and review within each case. Each radius represents a component of the CB-MPDSR approach including community coverage, reporting of maternal deaths, the conduct of maternal death verbal autopsies, reporting of perinatal deaths, and the conduct of perinatal death verbal autopsies. The length of each radius is proportional to key informants’ perceptions of the implementation scale of each component using a qualitative ranking of: 0 = none, 1 = some, 2 = many, and 3 = most. Data points on each radius are connected and color coded by case to represent the scale of implementation of CB-MPDSR approaches, with the largest pentagon outlined representing large scale implementation of CB-MPDSR

Penetration or how CB-MPDSR approaches are scaled, was assessed at the community, health system, and national levels (Supplementary Information: Table 1). The scale of CB-MPDSR approaches within communities was assessed in terms of the perceived coverage of communities and conduct of mortality reporting and review processes (Fig. 1). For maternal deaths, CXB and Uganda reported the highest scale of implementation. Nonetheless, across all cases, perinatal death reporting and review (verbal autopsies) was limited or not implemented at all.

In all cases, CB-MPDSR approaches were linked with facility-based MPDSR interventions. In Uganda and South Sudan, adoption of CB-MPDSR approaches were built upon existing facility-based approaches in efforts to scale up MPDSR implementation (in alignment with global guidance that recommends phased implementation starting at high-volume health facilities and scaling within lower levels of the health system before implementing at the community level [4, 6]). In Yemen, CB-MPDSR approaches were designed and implemented from program onset alongside facility-based approaches. However, CXB is an outlier in this regard; CB-MPDSR approaches were initiated first and used as foundation for development of a facility-based MDSR system. At the time of program design, health facilities were supported and ran by a multitude of international and local implementing agencies in CXB – with separate reporting systems, hierarchical structures, and sometimes with multiple partners offering different services within the same health facility. Meanwhile, a vast community health worker network (> 1400 CHWs) capable of conducting a weekly household census in the camps was supported by UN agencies and well-coordinated by an inter-agency CHW working group.

At the national level, only 2 of 4 cases (CXB and Uganda) had national MPDSR systems in place. In Ugandan refugee settlements, both community- and facility-based MPDSR approaches were integrated with the national MPDSR systems; yet, CB-MPDSR approaches were not implemented at a national scale outside of refugee settlements. Conversely, the national Bangladeshi MPDSR system and the CXB MDSR system were not linked or integrated to any extent.

#### Fidelity

Fidelity, the degree by which CB-MPDSR approaches were implemented as intended was also assessed and summarized through the exploration of four main constructs: adherence to established implementation processes, implementing actor responsiveness, quality of death reporting and review, and community engagement (Supplementary Information: Table 2).

Adherence to CB-MPDSR processes was variable. Strong community-based identification, reporting, and conduct of verbal autopsies of maternal deaths was reported in CXB, while Uganda, South Sudan, and Yemen reported significant delays, under-reporting of maternal deaths, and limited administration of verbal autopsies. Among partners implementing community-based approaches for perinatal deaths in Uganda and South Sudan, adherence to identification, notification, and review processes for these deaths was disproportionately low. Nonetheless, cases were universally affected by challenges in addressing identified recommendations from verbal autopsies, reporting either weak and/or no response implementation due to financial constraints.

Fidelity of CB-MPDSR approaches depended on a range of community actors. In all cases, apart from Yemen, implementation was led primarily by community health worker cadres (CHWs, VHTs, Boma health workers, or home health promoters) linked to the closest health facility. However, active participation in the reporting and conduct of verbal autopsies among these actors were only reported within higher-resourced refugee contexts (CXB and Uganda). In Yemen, death reporting from funeral personnel was ad hoc, dependent upon the willingness of each individual to consistently participate.

Overall, the quality of community-based reporting and review of maternal and perinatal deaths was low. Across all contexts, delays in reporting deaths and conducting verbal autopsies often exacerbated recall bias among families and caregivers yielding limited information to understand what happened to deceased mothers and babies within the community. Poor documentation of patient care and limited access to patient records to triangulate with family accounts also aggravated difficulties in determining the underlying causes or determinants of maternal and perinatal deaths.

However, despite reported issues with adherence to CB-MPDSR implementation processes, evidence of ongoing community engagement was shared in all contexts, including participation of community leadership in awareness and orientation sessions, verbal autopsy teams, and dialogues to discuss implications of community maternal and perinatal deaths. Some partners implementing MPDSR in Ugandan refugee settlements have also created community-led accountability committees to ensure active participation, buy-in, and input from community members.

### Factors influencing implementation of CB-MPDSR approaches

Two emerging themes were identified as primary factors that influenced implementation of CB-MPDSR approaches: (1) implementation inputs and (2) socio-cultural dynamics.

#### Implementation inputs

CB-MPDSR approaches across the diverse crisis-affected contexts hinged on the availability of financial and human resources to effectively implement MPDSR and related death review interventions at the community level. In South Sudan and Yemen where weakened health systems significantly depend upon external funding, limited and short-term humanitarian funding often did not trickle down to support CB-MPDSR approaches, and rather was prioritized elsewhere (e.g., lifesaving health service delivery) to meet immediate needs of the population. Even in the better-resourced refugee contexts of CXB and Uganda, funding cuts and shortages greatly impacted household coverage and capacity to conduct verbal autopsies. Across settings, financial constraints prevented programs from adequately training, equipping, and compensating community actors and health workers, in addition to limiting community engagement to mount adapted responses. Community health worker cadres as all other health providers, were often under-paid and overburdened by competing programmatic expectations and impractical catchment areas. Vacillating funding and low compensation also heightened attrition among community health worker cadres and trained health providers responsible for verbal autopsies looking for stability and/or career advancement.

#### Socio-cultural dynamics

The level of adoption, penetration, and fidelity to CB-MPDSR approaches were a product of transparency, trust, and communication between communities, health systems, and humanitarian actors, or lack thereof. In these humanitarian contexts where many populations were afflicted with ethnic marginalization, socio-cultural divisions, or conflict, established trust between community members and implementers of CB-MPDSR approaches was crucial for successful implementation. Early engagement via awareness or sensitization sessions to introduce CB-MPDSR enhanced transparency and built buy-in from community leadership. Selection of community health worker cadres originating from the communities where they worked and/or inclusion of actors well-versed in the communities’ norms also improved participation in reporting and review of community deaths. These actors were able to mediate internal community tensions, mitigate heightened sensitivities rooted in hostility and blame, and directly address misunderstandings between communities and health system actors.

*“Surveillance officers are well known in the communities which facilitates access and acceptance for reporting and review; [it’s] easier to garner trust.”* -Key informant in Yemen

*“Community members were more upfront with Rohingya volunteers [CHWs], compared to Bangladesh volunteers [CHWs]. There appears to be a lot of bias.”* -Key informant in CXB

*“Because these are very emotional processes… you are asking the family members, or they are there, seated there and the health workers are describing what happened. It becomes difficult for them to take. So, most of the time we work with the community health workers that live with them… so they know this family very well or they know their mother very well.”* -Key informant in South Sudan

*“We have the traditional birth attendant who helped deliver the newborn present during that verbal autopsy interview. Both to help with data recall and to answer some of the delivery questions. And also, to really start to establish a line of communication between the NGOs or the community-based organizations and the traditional birth attendants in the community.”* – Key informant in CXB

Some key informants also shared how cultural misconceptions, secrecy, and superstitions related to the cause or determinants of deaths encumbered reporting of maternal and perinatal mortality at the community level. Even so, CHWs and other supporting actors intimately familiar with the communities where they work were able to garner trust and navigate the socio-cultural factors influencing disclosure of maternal and perinatal deaths.

*“People are superstitious so if somebody starts seizing, they don't think ‘Oh, this is eclampsia, and we need to give her magnesium sulfate.’ They have a whole storyline around supernatural phenomena that might be going on which may be totally unrelated to the pregnancy. Their paradigm of reality in terms of being able to identify who in your community died from a pregnancy related complication in the last week is harder and they also might hide pregnancies.”* – Key informant in CXB

*“Sometimes when this [maternal and perinatal mortality] happens in the community, the mothers tend to keep quiet, especially in an event of a perinatal death. Especially for stillbirth, it is difficult most of the time to get this information from them. Unless it occurs among the TBA or midwives, that is when we can get this information. Otherwise, they tend to stay quiet about it, and that is a challenge that affects access to this information.”* – Key informant in South Sudan

CB-MPDSR approaches employing active surveillance via community health worker cadres were most effective in navigating these complex socio-cultural dynamics. In refugee contexts or where populations had little mobility, active surveillance delivered by CHWs with assigned household blocks strengthened rapport with families and minimized under-reporting, especially for perinatal deaths, as not only deaths but pregnancies could be consistently monitored.

Yet, communities reliant upon humanitarian aid were often reluctant to identify and share detailed information about deaths. Key informants shared that families may not report deaths to humanitarian actors when rations distributed and/or services provided are based on (or perceived to be based on) household size. Even when reassured that CB-MPDSR approaches are not connected to ration distribution or aid calculations, fear that humanitarian actors will still find out and/or someone else will hear or report on their behalf paralyzes some communities.

*“When a child is born into a family, they get registered into the family and so it increases the family size and therefore an increase in the amount of food rations they are entitled to from UNHCR or from the UN, and so, if you declare that probably someone has passed away, it kind of communicates that the family size has reduced, and therefore the benefits in terms of the food rations and the supplies they get will also be a bit lower. So sometimes, for such reasons, people are a bit reserved in declaring the deaths in the communities to us.”* – Key informant in Uganda

*“We are speaking about a place where in 10 square feet there are two or three houses. These are very close informal settlements. If I speak to you like this, three other houses can hear me. So confidential big issue… Like I want to speak about my wife's death but if I say that my wife was pregnant, she was full term…Maybe if this information goes out and if the people were talking about my information... They also get food rations from UNFPA and other humanitarian actors. So they feel if it is known that someone in my family has died, the rations will go from 18 eggs, if they get 6 eggs per person when there was 3 persons and now one person died. Confidentiality is very important, and this information should not be out somewhere.”* – Key informant in CXB

In addition, fluctuations in instability and the advent of environmental disasters impede maternal and perinatal death reporting and verbal autopsies due to population movement or displacement and community inaccessibility. Programs have equipped community actors with mobile technology to report deaths and conduct adapted and shortened verbal autopsies with family or community members via phone as an interim measure. However, reliability and credibility of data are questionable.

To improve coverage of community-based maternal and perinatal death reporting, some partners have trained other community actors (e.g., traditional birth attendants, community-midwives, funeral personnel). However, this approach often required more resources, supervision, and adaptation of reporting tools for lower literacy levels to ensure appropriate classification and documentation of maternal and perinatal death cases at an unknown cost–benefit in terms of financial resources and data quality. For example, traditional birth attendants (TBAs) have also been used to report and support verbal autopsies of maternal and perinatal deaths in Uganda and South Sudan; but, due to recent legislation prohibiting TBAs from attending deliveries, their participation in CB-MPDSR approaches and the participation of the communities in which they serve have reduced and/or stopped.

*“They [traditional birth attendants] fear reporting deaths when it occurs in their custody because the government has outlawed traditional birth attendants. They still conduct the deliveries illegally, so when a mother dies in their custody, they tend to not want to report that death to the authorities for fear of reprisal or being victimized... Some deaths go unreported because these occurred at the home of a traditional birth attendant and everyone around that village does not want to report it because the TBA might be victimized or blamed for that.”* – Key informant in South Sudan

*“Many times, we have seen these things [low reporting of maternal and perinatal deaths] happening with the traditional birth attendants… In Uganda, it is illegal for traditional birth attendants to deliver mothers, so they know the consequences in case a mother dies in their hands.”* -Key informant in Uganda

## Discussion

This study elucidates the on-the-ground realities of CB-MPDSR approaches in diverse humanitarian contexts. Adoption of CB-MPDSR interventions, penetration within communities and health systems and fidelity to established implementation processes varied widely, with stark differences between refugee and other crisis-affected contexts. Nonetheless, our findings provide the first in-depth analysis of factors influencing implementation of CB-MPDSR approaches across crisis-affected settings.

Under-reporting of maternal and perinatal mortality at the community level was universally declared, even when higher-resourced active pregnancy and mortality surveillance systems were in place. Similar to previous studies, key informants shared obstacles in community-based identification and notification of maternal and perinatal deaths related to population migration [21], limitations in geographical access due to insecurity or natural disasters [15, 18, 21], socio-cultural norms or disincentives [19, 25], and mistrust in the health system or implementing actors [15, 19, 21, 27]. Conduct of verbal autopsies by medical professionals or teams were also challenged by financial and human resource restraints and insufficient, incomplete, or misleading patient data.

Nevertheless, introduction or scale up of CB-MPDSR approaches in humanitarian settings should not be deterred by such bottlenecks. While highly sensitive prospective community-based surveillance systems may be ideal for timely mortality measurement [65–67], the value of CB-MPDSR approaches lies in understanding why mothers and babies are dying. Given the context and ever-changing funding landscape, this may be achieved through the report and review of only a sample of maternal and perinatal deaths occurring within communities. Similarly, issues with timeliness of reporting and review can be addressed by allowing for flexibility in the systems [68]. In crisis-affected contexts where a vast number of births and maternal and perinatal deaths occur at home undocumented by health systems, CB-MPDSR approaches provide a spotlight on important community and health system factors that impact the survival of the world’s most vulnerable populations.

Each CB-MPDSR approach provides unique advantages when developing systems within humanitarian contexts. Impactful implementation, thus, depends on adaptation and contextualization of CB-MPDSR strategies given available financial and human resources, as well as levels of security, and established trust and communication between communities and health programs. Within higher-resourced contexts with well-defined health service catchment areas (e.g., refugee or IDP camps), active community-based surveillance executed through regular household visits by community health cadres may be more feasible and render more robust information at the population level. Conversely, contexts with less resources may consider passive surveillance approaches relying on community actors to report deaths to the health facility or implementing agency. We have found that diversification of community-based implementing actors (e.g., inclusion of TBAs, morgue and burial attendants) improves the penetration of CB-MPDSR approaches. As in other studies, recruitment of actors to work within the communities where they reside imparts great benefit in terms of community acceptability and buy-in and assists in overcoming socio-cultural barriers [59, 69, 70]. While well noted in our study and by the evidence base to come with their own set of challenges [15], building the capacity of TBAs for death notification (as well as community midwifes) could provide access to the most hard-to-reach communities in humanitarian contexts.

To better understand effectiveness and how to maximize adaptations of CB-MPDSR approaches, additional implementation science and operational research is needed. Considerations of CB-MPDSR approaches not reported by key informants in our study are also warranted, namely the training of additional community actors (e.g., village administrators, religious affiliates, etc.), integration with local civil registration and vital statistics and other administrative systems, and use of mobile clinics. Given CXB’s high fidelity and penetration of CB-MDSR, which subsequently supported development of facility-based systems, further exploration of the feasibility and effectiveness of independent CB-MPDSR approaches in other humanitarian settings is also worth studying.

The case study is not without limitations. Of note, social autopsies were not reported in any of the contexts. However, as Thomas et al. highlight, the nomenclature utilized may not have been aligned with definitions and conceptualization of social autopsy methodology [59]. In descriptions of CB-MPDSR approaches, reference was often made to the social determinants of factors attributable to death. Nevertheless, upon subsequent inquiry, key informants did not feel that verbal autopsies conducted within their respective programs had social autopsy components (i.e., assessed social contributors to death). Systematic review of all autopsy tools would have shed further insight but were not available to the research team. In addition, findings from our study are limited to qualitative inquiries in four diverse humanitarian contexts and may not be generalizable to all programs employing CB-MPDSR approaches across these settings globally. Travel restrictions during the COVID-19 pandemic only allowed for virtual interviews, which may have limited the sample of key informants to those with access to the internet. Also, power dynamics between the interviewer and participants or between participants during group interviews may have created social desirability bias. MM actively tried to mitigate these effects by practicing ongoing reflexivity and ensuring participants had equitable opportunity to contribute during group interviews. We employed a health systems lens to this study and focused on selecting implementing actors as key informants. However, future research should also consider documenting community leader and member viewpoints to complement health and humanitarian actor perspectives and elevate voices often underrepresented in research [71].

## Conclusion

CB-MPDSR approaches offer insights into important systemic and cultural factors contributing to mortality within humanitarian settings. Our results call for more investment in understanding how to effectively implement CB-MPDSR to strengthen community-health system dynamics and improve the quality of maternal and neonatal health service delivery in crisis affected contexts. Development of operational guidance to assist humanitarian actors in introducing or bolstering both active and passive CB-MPDSR approaches is crucial, so as to support a system reflective of complex realities faced by these diverse and mobile communities.

## Supplementary Information

Below is the link to the electronic supplementary material.

Supplementary Material 1.

## Data Availability

The datasets used and/or analyzed during the current study are available from the corresponding author on reasonable request.

## Abbreviations

CB-MPDSR: Community-based maternal and perinatal death surveillance and response;
CBS: Community-based surveillance
CHW: Community health workers;
CXB: Cox’s bazar;
EWARS: Early warning alert and response system;
FY: Fiscal year;
IAWG: Inter-agency working group on reproductive health in crises;
IDP: Internally displaced persons;
INGO: International non-governmental organization;
LMIC: Low- and middle-income countries;
MDSR: Maternal death surveillance and response;
MPDSR: Maternal and perinatal death surveillance and response;
NGO: Non-Governmental Organization;
TBA: Traditional birth attendant;
VHT: Village health teams;
WHO: World Health Organization;
UN: United Nations;
UNFPA: United Nations Population Fund;
UNHCR: United Nations High Commissioner for Refugees;
UNOCHA: United Nations Office for Coordination of Humanitarian Affairs

## References

[1] Beyond the Numbers: Reviewing maternal deaths and complications to make pregnancy safer. Geneva, Switzerland: World Health Organization; 2004.

[2] Every Newborn: an action plan to end preventable deaths. Geneva: World Health Organization and UNICEF; 2014.

[3] Strategies toward ending preventable maternal mortality (EPMM). Geneva, Switzerland: World Health Organization; 2015.

[4] Maternal Death Surveillance and Response: Technical Guidance. Geneva, Switzerland: World Health Organization; 2013.

[5] Count MEB. Audit and review of stillbirths and neonatal deaths. Geneva, Switzerland: World Health Organization; 2016.

[6] Maternal and Perinatal Death Surveillance and Response: Materials to Support Implementation. Geneva, Switzerland: World Health Organization; 2021.

[7] Kinney MV, Walugembe DR, Wanduru P, Waiswa P, George A. Maternal and perinatal death surveillance and response in low- and middle-income countries: a scoping review of implementation factors. Health Policy Plan. 2021. 10.1093/heapol/czab011. 33712840 10.1093/heapol/czab011 PMC8227470

[8] Pattinson R, Kerber K, Waiswa P, Day LT, Mussell F, Asiruddin S, et al. Perinatal mortality audit: counting, accountability, and overcoming challenges in scaling up in low- and middle-income countries. Int J Gynecol Obstet. 2009;107(SUPPL):S113–22. 10.1016/j.ijgo.2009.07.011 19815206

[9] Kerber KJ, Mathai M, Lewis G, Flenady V, Erwich JJH, Segun T, et al. Counting every stillbirth and neonatal death through mortality audit to improve quality of care for every pregnant woman and her baby. BMC Pregnancy Childbirth. 2015;15(Suppl 2):S9. 26391558 10.1186/1471-2393-15-S2-S9 PMC4577789

[10] Lusambili A, Jepkosgei J, Nzinga J, English M. What do we know about maternal and perinatal mortality and morbidity audits in sub-Saharan Africa? A scoping literature review. Int J Hum Rights Healthc. 2019;12(3):192–207.

[11] Mary M, Tappis H, Scudder E, Creanga AA. Implementation of maternal and perinatal death surveillance and response and related death review interventions in humanitarian settings: a scoping review. J Glob Health. 2024;14:04133. 38991208 10.7189/jogh.14.04133 PMC11239189

[12] OCHA. Humanitarian Action: Analysing Needs and Response. 2023. Global Humanitarian Overview 2023. Available from: https://hum-insight.info/

[13] Meeting TC to the J 2018 W. A definition for community-based surveillance and a way forward: results of the WHO global technical meeting, France, 26 to 28 June 2018. Eurosurveillance. 2019 Jan 10;24(2):1800681. 10.2807/1560-7917.ES.2019.24.2.1800681 PMC6337056 30646977

[14] Kobeissi L, Nair M, Evers ES, Han MD, Aboubaker S, Say L, et al. Setting research priorities for sexual, reproductive, maternal, newborn, child and adolescent health in humanitarian settings. Confl Health. 2021;15(1):1–10. 33771212 10.1186/s13031-021-00353-w PMC7995567

[15] Russell N, Tappis H, Mwanga JP, Black B, Thapa K, Handzel E, et al. Implementation of maternal and perinatal death surveillance and response (MPDSR) in humanitarian settings: insights and experiences of humanitarian health practitioners and global technical expert meeting attendees. Confl Health. 2022;16(1):1–16. 35526012 10.1186/s13031-022-00440-6 PMC9077967

[16] Proctor E, Silmere H, Raghavan R, Hovmand P, Aarons G, Bunger A, et al. Outcomes for implementation research: conceptual distinctions, measurement challenges, and research agenda. Adm Policy Ment Health. 2011;38(2):65–76. 20957426 10.1007/s10488-010-0319-7 PMC3068522

[17] Mary M, Tappis H, Scudder E, Creanga AA. Implementation of Maternal and Perinatal Death Surveillance and Response and related death review interventions in humanitarian settings: A Scoping Review. J Glob Health. in press. 10.7189/jogh.14.04133 PMC11239189 38991208

[18] Umbeli T, El Tahir S, Allah S, Saliheen N, Kunna A, Ahmed S, et al. Achievements and challenges of implementing maternal death reivew (MDR) in Sudan 2010–2015. Int J Curr Res. 2017;9:45139–43.

[19] Amsalu R, Costello J, Hasna Z, Handzel E. Estimating stillbirth and neonatal mortality rate among Rohingya refugees in Bangladesh, September 2017 to December 2018: a prospective surveillance. BMJ Glob Health. 2022;7(4):e008110. 35443939 10.1136/bmjgh-2021-008110 PMC9024274

[20] Bulletin N1 de la surveillance des deces maternels et riposte (SDMR): Janvier-Juin 2018 en Republique Democratique du Congo. UNFPA and WHO; 2018.

[21] Jarrett P, Zadravecz FJ, O’Keefe J, Nshombo M, Karume A, Roberts L. Evaluation of a population mobility, mortality, and birth surveillance system in South Kivu, Democratic Republic of the Congo. Disasters. 2020;44(2):390–407. 31231822 10.1111/disa.12370 PMC7154676

[22] Shittu O, Kinney M. Assessment of Maternal and Perinatal Death Surveillance and Response Implementation in Nigeria. Nigeria; 2017.

[23] Maternal Death Surveillance and Response: Inventory of Current Practices in Protracted Humanitarian Settings Supported by UNFPA. UNFPA; 2020.

[24] van Boekholt TA, Moturi E, Hölscher H, Schulte-Hillen C, Tappis H, Burton A. Review of maternal death audits in refugee camps in UNHCR East and Horn of Africa and Great Lakes Region, 2017–2019. Int J Gynaecol Obstet. 2023;160(2):483–91. 36217727 10.1002/ijgo.14504 PMC10092615

[25] Maternal and Neonatal Death Surveillance and Response (MNDSR) Process Evaluation. Baltimore, Maryland: Jhpiego; 2019.

[26] Umbeli T. Implementing maternal death surveillance and response (MDSR) in Sudan 2014–2017: achievements and challenges. Int J Gynaecol Obstet. 2018;143(S3):103.

[27] Boetzelaer EV, Chowdhury S, Etsay B, Faruque A, Lenglet A, Kuehne A, et al. Evaluation of community based surveillance in the Rohingya refugee camps in Cox’s Bazar, Bangladesh, 2019. PLoS ONE. 2020;15(12):e0244214. 33362236 10.1371/journal.pone.0244214 PMC7757896

[28] Handzel E, Zack R, Biswas A, Chowdhury S. Establishment of a community-based maternal mortality surveillance system among the Rohingya refugee population in Bangladesh. 2021.

[29] Maternal and Perinatal Mortality Surveillance and Response (MPMSR) in Rohingya Refugees camps in Cox’s Bazar, Bangladesh: Annual Report 2020. Bangladesh: UNFPA Bangladesh; 2020.

[30] Map of 2023 Countries with UN Humanitarian Appeals that Contribute to Global Maternal Deaths, Newborn Death and Stillbirths. IAWG Sub-Working Group on Maternal and Newborn Health; 2023.

[31] Global Sub-Working Group dedicated to MPDSR in Humanitarian Settings. Implementation of Maternal and Perinatal Death Surveillance and Response (MPDSR) Interventions in Humanitarian Settings: A Landscape Analysis. Baltimore, Maryland; 2021.

[32] Data [Internet]. 2025. World Bank Country and Lending Groups. Available from: https://datahelpdesk.worldbank.org/knowledgebase/articles/906519-world-bank-country-and-lending-groups

[33] FY23 List of Fragile and Conflict-affected Situations [Internet]. World Bank; 2023. Available from: https://thedocs.worldbank.org/en/doc/9b8fbdb62f7183cef819729cc9073671-0090082022/original/FCSList-FY06toFY22.pdf

[34] AlignMNH, Interagency Working Group on Reproductive Health in Crises, Jhpiego. MNH Targets, Measurement, & Data. 2022. Maternal and Neonatal Mortality in Humanitarian Settings Dashboard. Available from: https://www.alignmnh.org/issue/mnh-targets-measurement-and-data/

[35] Trends in maternal mortality 2000 to 2020: Estimates by WHO, UNICEF, UNFPA, World Bank Group and UNDESA/Population Division. Geneva: World Health Organization; 2023.

[36] United Nations Inter-agency Group for Child Mortality Estimation (UN, IGME). Levels & Trends in Child Mortality: Report 2022, Estimates developed by the United Nations Inter-agency Group for Child Mortality Estimation. New York: United Nations Children’s Fund; 2023.

[37] United Nations Inter-agency Group for Child Mortality Estimation (UN IGME). Never Forgotten: The situation of stillbirth around the globe. New York: United Nations Children’s Fund; 2023.

[38] Uganda: The Refugees Regulations. 2010.

[39] Uganda: The Refugee Act 2006 [Internet]. Act 21 May 24, 2006. Available from: https://www.refworld.org/docid/4b7baba52.html

[40] Inter-Agency Uganda Country Refugee Response Plan: 2022–2025. United Nations High Commissioner for Refugees (UNHCR); 2022.

[41] Operational Update: Uganda 1–30 June 2022. Uganda: UNHCR; 2022 Aug.

[42] Burnham GM, Rowley EA, Ovberedjo MO. Quality design: a planning methodology for the integration of refugee and local health services, West Nile, Uganda. Disasters. 2003;27(1):54–71. 12703152 10.1111/1467-7717.00219

[43] Joint Response Plan. Rohingya Refugee Humanitarian Crisis. Bangladesh: Inter Sector Coordination Group; 2023.

[44] Refugee Influx Emergency Vulnerability Assessment (REVA-5): Technical Report. Bangladesh: World Food Programme; 2022 Jun.

[45] Health Sector Cox’s Bazar Monthly Bulletin: November-December 2022. Cox’s Bazar, Bangladesh: World Health Organization; 2022.

[46] Humanitarian Response Plan: South Sudan. South Sudan: OCHA; 2023.

[47] Humanitarian Outcomes Aid Worker Security Database [Internet]. Available from: https://aidworkersecurity.org/incidents

[48] Gianaris K, Atem J, Chen AP, Chang AH, Russell A, Hsu EB. Providing quality of care in fragile and vulnerable settings: lessons from South Sudan. Ann Glob Health. 2021;87(1):126. 35036333 10.5334/aogh.3506 PMC8698219

[49] Berendes S, Lako RL, Whitson D, Gould S, Valadez JJ. Assessing the quality of care in a new nation: South Sudan’s first national health facility assessment. Trop Med Int Health. 2014;19(10):1237–48. 25134414 10.1111/tmi.12363

[50] Jones A, Howard N, Legido-Quigley H. Feasibility of health systems strengthening in South Sudan: a qualitative study of international practitioner perspectives. BMJ Open. 2015;5(12):e009296. 26700280 10.1136/bmjopen-2015-009296 PMC4691708

[51] Erismann S, Gürler S, Wieland V, Prytherch H, Künzli N, Utzinger J, et al. Addressing fragility through community-based health programmes: insights from two qualitative case study evaluations in South Sudan and Haiti. Health Res Policy Syst. 2019;17(1):20. 30764847 10.1186/s12961-019-0420-7 PMC6376698

[52] Lutwama GW, Sartison LJ, Yugi JO, Nehemiah TN, Gwang ZM, Kibos BA, et al. Health services supervision in a protracted crisis: a qualitative study into supportive supervision practices in South Sudan. BMC Health Serv Res. 2022;22(1):1–16. 36242016 10.1186/s12913-022-08637-4 PMC9568951

[53] Evaluation of the South Sudan Health Pooled Fund. Integrity; 2018.

[54] Global Report on Internal Displacement (GRID) 2022. Geneva: The Internal Displacement Monitoring Center; 2022.

[55] Humanitarian Response Plan Yemen. OCHA; 2023.

[56] Global Peace Index 2022: Measuring Peace in a Complex World. Sydney: Institute for Economics & Peace; 2022 Jun.

[57] Humanitarian Needs Overview Yemen. Yemen: OCHA; 2023.

[58] Proctor E. Implementation research in mental health services: an emerging science with conceptual, methodological, and training challenges. Adm Policy Ment Health [Internet]. 2009 Jan [cited 2021 Mar 19];36(1). Available from: http://pubmed.ncbi.nlm.nih.gov/19104929/10.1007/s10488-008-0197-4 PMC3808121 19104929

[59] Thomas LM, D’Ambruoso L, Balabanova D. Use of verbal autopsy and social autopsy in humanitarian crises. BMJ Glob Health. 2018;3(3):e000640. 29736275 10.1136/bmjgh-2017-000640 PMC5935165

[60] Vaismoradi M, Jones J, Turunen H, Snelgrove S. Theme development in qualitative content analysis and thematic analysis. J Nurs Educ Pract. 2016;6(5):100.

[61] Yin RK. Case Study Research and Applications: Design and Methods. Sixth. SAGE Publications; 2017. 346 p.

[62] Olmos-Vega FM, Stalmeijer RE, Varpio L, Kahlke R. A practical guide to reflexivity in qualitative research: AMEE Guide No. 149. Med Teach. 2023 Mar 4;45(3):241–51. 10.1080/0142159X.2022.2057287 35389310

[63] Maternal and Perinatal Death Surveillance and Response Guidelines. Uganda: Reproductive Health Division, Ministry of Health; 2017.

[64] Maternal Mortality Audit National Guidelines. Yemen: Reproductive Health and Population Programme, Ministry of Public Health and Population; 2013.

[65] Checchi F, Roberts L. Interpreting and using mortality data in humanitarian emergencies: a primer for non-epidemiologists. London: Overseas Development Institute; 2005. (Network Paper). Report No.: 52.

[66] Fatusić Z, Kurjak A, Grgić G, Tulumović A. The influence of the war on perinatal and maternal mortality in Bosnia and Herzegovina. J Matern-Fetal Neonatal Med Off J Eur Assoc Perinat Med Fed Asia Ocean Perinat Soc Int Soc Perinat Obstet. 2005;18(4):259–63. 10.1080/147670500198501 16318977

[67] Purdin S, Spiegel P, Mack KP, Millen J. Surveillance beyond camp settings in humanitarian emergencies: findings from the humanitarian health information management working group. Prehosp Disaster Med. 2009;24(SUPPL.2):s202–5. 19806541 10.1017/s1049023x00021592

[68] van Boekholt TA, Moturi E, Hölscher H, Schulte-Hillen C, Tappis H, Burton A. Review of maternal death audits in refugee camps in UNHCR East and Horn of Africa and Great Lakes Region, 2017–2019. Int J Gynaecol Obstet. 2022. 10.1002/ijgo.14504. 36217727 10.1002/ijgo.14504 PMC10092615

[69] Bowden S, Braker K, Checchi F, Wong S. Implementation and utilisation of community-based mortality surveillance: a case study from Chad. Confl Health. 2012;6(1):11. 23186330 10.1186/1752-1505-6-11 PMC3560199

[70] Spiegel PB, Sheik M, Woodruff BA, Burnham G. The accuracy of mortality reporting in displaced persons camps during the post-emergency phase. Disasters. 2001;25(2):172–80. 11434236 10.1111/1467-7717.00169

[71] Lokot M. Whose Voices? Whose Knowledge? A Feminist Analysis of the Value of Key Informant Interviews. Int J Qual Methods. 2021;1(20):1609406920948775.

