# Supplementary material for: Community-based maternal and perinatal death surveillance and response: a comparative case study of implementation realities from humanitarian contexts
Source: BMC Public Health. 2025 Oct 1;25:3256. doi: 10.1186/s12889-025-24440-2 (PMC12486539; doi:10.1186/s12889-025-24440-2)
Supplement: Supplementary file 1 — Supplementary Material 1. [file 12889_2025_24440_MOESM1_ESM.pdf]

Supplementary Information:

Annex 1: Interview Guide

PARTICIPANT ROLE

- Please tell me a bit about yourself –what is your current position at [agency]?
- What is your current role in implementing or providing support to the CB-MPDSR in [location]?
- What is the scale of implementation?
  - How many camps/health facilities/communities in [location]?
  - On average, how many actors [health providers, community health workers, etc.] are involved in implementing CB-MPDSR ?

IMPLEMENTATION:

- Can you give a brief overview of how CB-MPDSR was conceived and developed?
  - Who identified the need for the intervention?
  - Who led the process/decided to implement the CB-MPDSR?
  - How did the humanitarian setting impact this decision?
- Did you pilot the program first? Please describe this process.
- How have you adapted CB-MPDSR to the humanitarian context? Please describe what adaptations were made, if any, and why.
- Has CB-MPDSR been implemented as planned? Why or why not? Please describe any deviation.
  - Probe for each step of the 4-step cycle (identify/notify, review, analyze/recommend, and respond)
- In your opinion, how is CB-MPDSR implemented in [location] different from the national system?
- In your opinion, what lessons learned from the national program could/should be applied to the CB-MPDSR in [location]?
- From your perspective, what are the main achievements of CB-MPDSR in [LOCATION] to date?
- What are your thoughts regarding the feasibility of expanding the system to perinatal or neonatal death surveillance?
  - What challenges have you faced?
  - Will there be opportunities to expand to perinatal death surveillance in the future?

RESOURCES

- To what extent are implementing partners and key actors engaged in CB-MPDSR?
  - To what extent is there local ownership of CB-MPDSR? Please describe.
- How is the coordination between the CB-MPDSR and other health information systems? How do they communicate with each other?
- Are there key stakeholder groups that should be involved, but are not? If yes, who and why do you think they are excluded?

- Are resources (financial and workforce) sufficient for implementation? Why or why not?
    - Probe: Agency resources v. government resources?
- Which funding sources are dedicated to CB-MPDSR?
- In your opinion, what are the biggest challenges in integrating CB-MPDSR with the national program and sustaining CB-MPDSR in the long term? How have these challenges been overcome?

FINAL STAKEHOLDER PERCEPTIONS

- What are key lessons learned from implementation of CB-MPDSR in [LOCATION]?
- What are your top recommendations for an implementing partner who is seeking to implement CB-MPDSR in a humanitarian context?
- Is there anything else you think we should know about implementation of CB-MPDSR in humanitarian settings in [LOCATION]? Anything important we've missed talking about?

Table 1: Penetration: The integration of CB-MPDSR interventions within communities and health systems in humanitarian settings, summarized by case

	Cox's Bazar refugee camps	Uganda refugee settlements	South Sudan	Yemen
**Scale**	See Figure 1			
**Linkages with facility-based MPDSR approaches**	• Facility-based MDSR built upon CB-MDSR approach<br>• Triangulation of community and facility-based reporting identifies unreported facility deaths	• CB-MPDSR approaches are expansion of Facility based-MPDSR interventions	• CB-MPDSR approaches are expansion of facility based-MPDSR interventions	• Facility – and community-based approaches are integrated
**Integration with national MPDSR systems**	• No integration with national MPDSR system	• Full integration with national MPDSR system	• No existing national MPDSR system	• No existing national MPDSR system
Notes:<br>*Abbreviations: CB-MPDSR: Community-based Maternal and Perinatal Death Surveillance and Response; MDSR: Maternal Death Surveillance and Response; MPDSR: Maternal and Perinatal Death Surveillance and Response*				

Table 2: Fidelity: The degree to which CB-MPDSR interventions were implemented as intended, according to local, national, or international guidelines or action plans, summarized by case

	**Cox's Bazar refugee camps**	**Uganda refugee settlements**	**South Sudan**	**Yemen**
**Adherence to CB-MPDSR intervention cycle or processes**	• Strong CB reporting of maternal deaths<br>• Delays in verbal autopsies<br>• Response needs strengthening	• Delays in notification and verbal autopsies of CB maternal deaths<br>• CB perinatal reporting and verbal autopsies is partner dependent and not mandated by UNHCR<br>• Response needs strengthening	• Delays in CB death notification and verbal autopsies<br>• Response needs strengthening	• CB reporting is ad hoc<br>• Delays in verbal autopsies<br>• Response not feasible due to financial constraints
**Quality of reporting and review**	• Incomplete and poor documentation of patient data<br>• Community hesitation to provide detailed information about the death<br>• Ensuring confidentiality of verbal autopsies often a problem given density and lack of privacy within the camps	• Incomplete and poor documentation of patient data<br>• Community hesitation to provide detailed information about the death	• Incomplete and poor documentation of patient data<br>• Community hesitation to provide detailed information about the death	• Incomplete and poor documentation of patient data<br>• Community hesitation to provide detailed information about the death
**Implementing actor responsiveness**	• CHWs actively report and participate in system<br>• CHW supervisors supportive of system for community-based supervision.<br>• Midwives actively conduct verbal autopsies	• VHT/CHWs actively report and participate in system<br>• TBAs and community members reluctant to report due to illegalization of TBAs and fear that TBA might be victimized or blamed for death	• CHW (Boma Health workers)/Home health promoter responsiveness and participation in system varies by partner<br>• TBAs and community members reluctant to report due to illegalization of TBAs and fear that TBA might be victimized or blamed for death	• Ad hoc reporting from funeral personnel (including body/corpse washers) and community members<br>• Active participation among surveillance officers
**Community engagement**	• Community and religious leaders involved in awareness sessions that target cultural and	• Active community involvement in sensitization sessions conducted to	• Active community involvement in sensitization sessions conducted to	• Active community involvement in sensitization sessions conducted to

	familial decision-makers for health care seeking	encourage reporting and participation in the system<br>• Community leadership participates in verbal autopsy teams	encourage reporting and participation in the system<br>• One partner established community health committees to hold the health system accountable and engage community leadership	encourage reporting and participation in the system<br>• Community leaders and authorities participate in training to introduce the system
Notes:<br>*Abbreviations: CB: Community-based; CB-MPDSR: Community-based Maternal and Perinatal Death Surveillance and Response; CHW: Community Health Worker; TBA: Traditional Birth Attendant; UNHCR: United Nations High Commissioner for Refugees; VHT: Village Health Team*				
